# Influence of the 5′-terminal sequences on the 5′-UTR structure of HIV-1 genomic RNA

**DOI:** 10.1038/s41598-021-90427-9

**Published:** 2021-05-25

**Authors:** Camille Michiko Obayashi, Yoko Shinohara, Takao Masuda, Gota Kawai

**Affiliations:** 1 Department of Life Science, Graduate School of Advanced Engineering, Chiba Institute of Technology, Tsudanuma, 2-17-1, Narashino-shi, Chiba, 275-0016 Japan; 2 Department of Life and Environmental Sciences, Graduate School of Engineering, Chiba Institute of Technology, Tsudanuma, 2-17-1, Narashino-shi, Chiba, 275-0016 Japan; 3 Department of Immunotherapeutics, Graduate School of Medical and Dental Sciences, Tokyo Medical and Dental University (TMDU), Yushima, 1-5-45, Bunkyo-ku, Tokyo, 113-8519 Japan

**Keywords:** NMR spectroscopy, RNA

## Abstract

The 5′-UTR of HIV-1 genomic RNA is known to form specific structures and has important functions. There are three 5′-terminal sequences, G1, G2 and G3, with different localizations in the cell and virion particles as well as different efficiencies in translation and reverse transcription reactions. In the present study, the structural characteristics of the joint region between the TAR and PolyA stems was analysed, and it was found that small differences in the 5′-terminus affect the conformational characteristics of the stem-loop structures. In the G1 form, the two stems form a coaxial stem, whereas in the G2 and G3 forms, the two stems are structurally independent of each other. In the case of the G1 form, the 3′-flanking nucleotides of the PolyA stem are included in the stable coaxial stem structure, which may affect the rest of the 5′-UTR structure. This result demonstrates that the local conformation of this functionally key region has an important role in the function of the 5′-UTR.

## Introduction

In the promoter of HIV-1 provirus DNA, each of three continuous G residues works as the transcription initiation site^1–3^. The RNA transcribed from each transcription initiation site is called G1 form, G2 form, or G3 form^1^. The G1-form is dominant in virus particles, and the G3 form is dominant in the cytosol of infected cells^1^. It has also been shown that the G2 and G3 forms are enriched in polysomes^2^. Furthermore, under physiological-like ionic conditions, the 5′-leader RNA in G1 form adopts a dimeric structure, whereas the 5′-leader RNA in the G2 or G3 form exists predominantly as a monomer^2^. Our previous work also showed that the number of G residues in the 5′-terminus affects the reverse transcription reaction^1^. These facts indicate that the function of HIV-1 RNA is controlled by a small difference in the 5′-terminal sequence^1,2^.

Recently, Brown et al. analysed the structures of the 5′-region of RNA transcripts by NMR spectroscopy^4^. For the G1 form transcript, the 5′-Cap structure was hidden to prevent interaction with eIF-4E^4^, which may be the reason that the G1-form transcript was not concentrated in the ribosome fraction. In contrast, the G2 and G3-form transcripts were suggested to adopt a more flexible conformation in the PolyA stem, which allows the formation of extended central stems, including U5 and DIS, preventing dimerization^4^. Although the global structures of the 5′-terminus of the transcripts were determined, it is important to analyse the effect of the number of G residues on the local structure of the 5′-terminus of the HIV-1 transcripts at the nucleotide level using suitable model RNAs.

**Figure 1:**
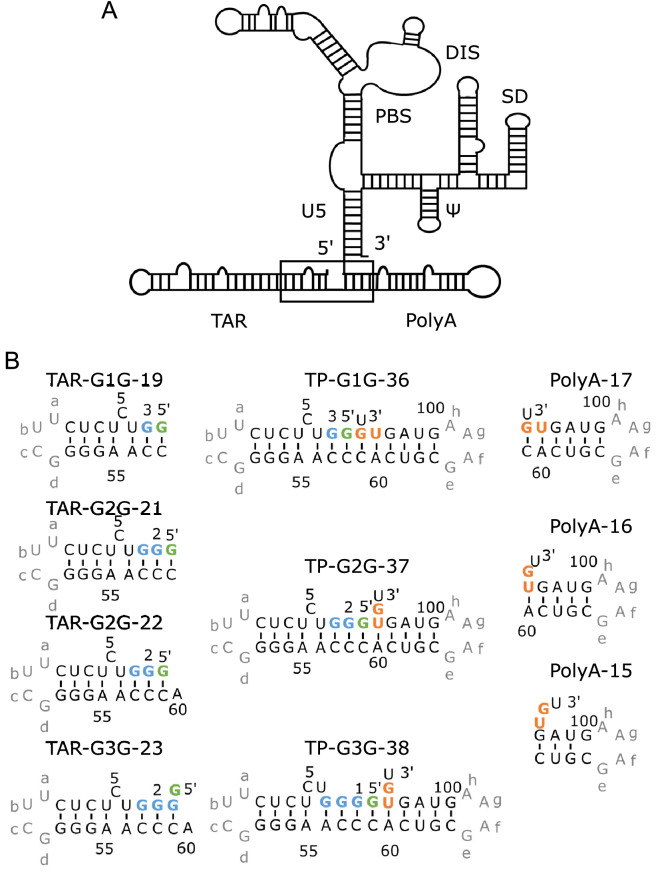
5′-UTR of the HIV-1 genomic RNA and design of model RNAs. (**A**) The secondary structure of the 5′-UTR was shown. The target of the present work is the region including the TAR and PolyA stems as indicated by a box. (**B**) The designed RNAs for the junction region are shown in the middle column. G1G, G2G and G3G were each fragmented around a junction of TAR-PolyA and both ends were capped by tetraloops; for TAR and PolyA, the UUCG and GAAA loops were added, respectively. These constructed residues were indicated by grey. The designed fragments were called TP-G1G-36, TP-G2G-37, and TP-G3G-38 according to the number of residues. Furthermore, these RNAs were divided into TAR and PolyA fragments, as shown in the left and right columns, so that possible base pairs remained for each fragment. TP-G1G-36 was divided into TAR-G1G-19 and PolyA-17. TP-G2G-37 was divided into TAR-G2G-21 and PolyA-16, or TAR-G2G-22 and PolyA-15. TP-G3G-38 was divided into TAR-G3G-23 and PolyA-15. The residue number starts with the first G residue of the three transcription start sites. The G residues corresponding to the three transcription start sites and 5′-Cap are coloured light blue and green, respectively. U104 and G105 at the 3′-end of the PolyA stem were coloured orange.

In the present study, we designed model RNAs corresponding to the junction region (Fig. 1A) and analysed the effect of the number of G residues at the 5′-terminus on their structures. G1-form RNA forms a stable structure with coaxial stacking between the two stems, whereas G2 and G3-form RNAs show weak interactions between the two stems, and the PolyA stem melts at lower temperatures. These findings strongly support the idea that the number of G residues at the 5'-terminus function in conformational regulation.

## Results

### Design of model RNAs for the three forms

The RNA fragments used for analyses are shown in Fig. 1B. The region shown by the box in Fig. 1A was extracted and the truncated ends for the TAR and PolyA stems were connected by the UUCG and GAAA sequences, respectively. In the native genomic RNA, the Cap structure exists in the 5′ end, however, in this study, a G residue was connected by the normal phosphodiester bond instead of the triphosphate group connecting the two 5′ hydroxy groups. The modelling analysis confirmed that the tertiary structures of the Cap structure with three methyl groups (m^7^GpppGmUm) and GGU were not different from each other (Supplemental Fig. S1). In addition, Brown et al. also showed that the properties were not different from each other^4^. For the three forms with different numbers of G residues, TP-G1G-36, TP-G2G-37 and TP-G3G-38 were designed.

**Figure 2:**
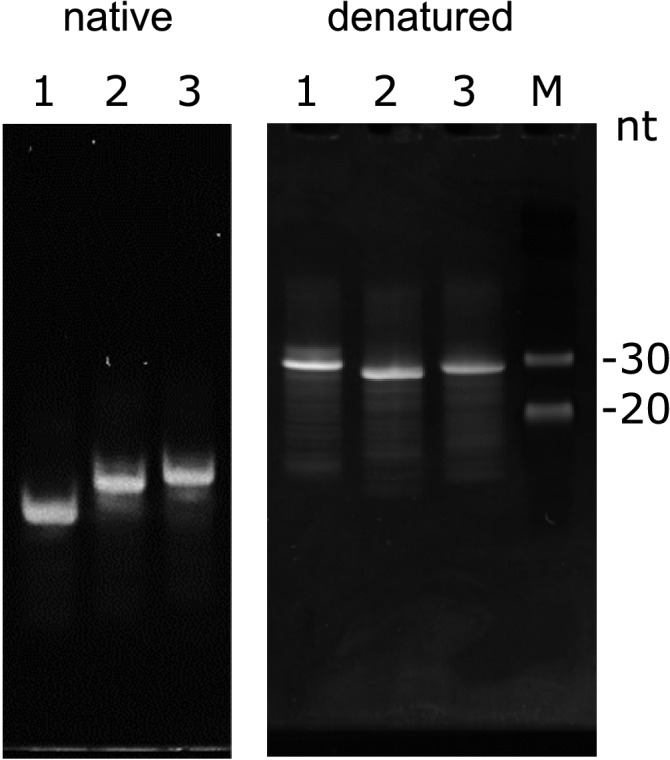
Characteristics of model RNAs analysed by PAGE. Twelve percent native (left) and denatured (right) PAGE are shown with TP-G1G-36, TP-G2G-37, and TP-G3G-38 for lanes 1, 2, and 3, respectively. Line M represents the size marker for single stranded RNAs.

Sub-fragments for each of the three fragments were also designed (Fig. 2B). For TP-G1G-36, the fragment was divided between C58 and C59 to form TAR-G1G-19 and PolyA-17. In the case of TP-G2G-37, two sets of fragments were designed: TAR-G2G-21 and PolyA-16 and TAR-G2G-22 and PolyA-15. For TP-G3G-38, the fragment was divided between A60 and C61 to form TAR-G3G-23 and PolyA-15.

### Property of the model RNA

To analyse the conformational properties of the three model RNAs, the electrophoretic mobilities of each were examined under the native conditions. The migration speed was faster for TP-G1G-36 than for the other two RNAs (Fig. 2), suggesting that the structure of TP-G1G-36 is different from the others. TP-G3G-37 and TP-G2G-38 showed similar migration speeds, suggesting that these RNAs resemble each other in structure. This can be explained by the fact that the structure of TP-G1G-36 is compact and migrates faster than the others. On the other hand, TP-G2G-37 and TP-G3G-38 migrate slowly, probably due to structural fluctuations. Under denaturing conditions, TP-G2G-37 and TP-G3G-38 showed faster migration, suggesting that the structures of the TAR stems in TP-G2G-37 and TP-G3G-38 were stable and only partially denatured in the presence of 7 M urea. Notably, these two RNAs migrated faster than the 30 nt marker.

**Figure 3:**
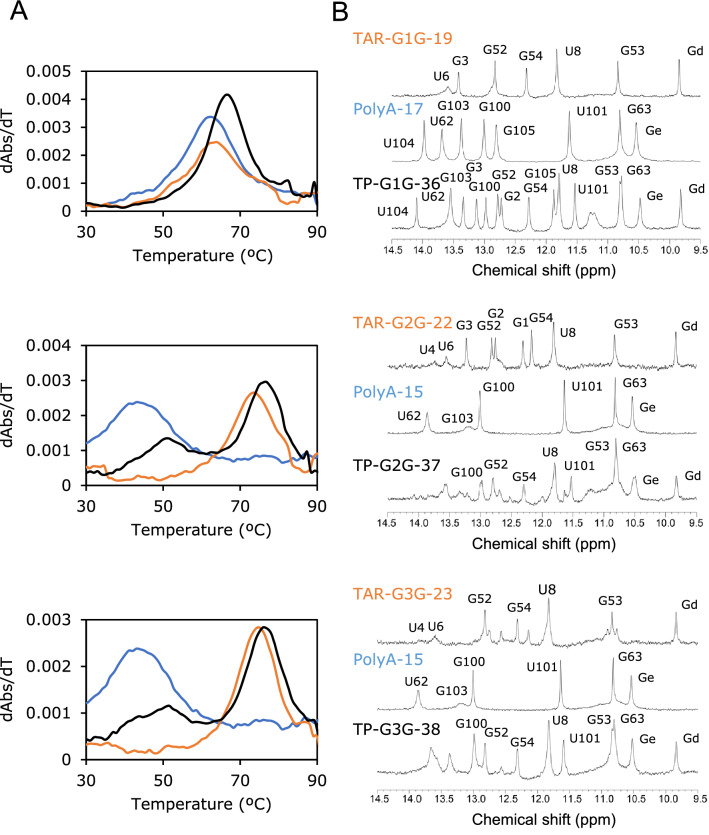
Characteristics of model RNAs analysed by thermal melting and NMR. (**A**) The first derivatives of UV melting curves for TP-G1G-36, TP-G2G-37, and TP-G3G-38 with the corresponding sub-fragments. The absorbance at 260 nm was shown in the temperature range of 30 to 90 °C. Model RNAs, TAR and Poly-A sub-fragments are coloured black, orange and blue, respectively. (**B**) Imino proton spectra measured at 283 K.

Then, the thermal melting profiles of the three RNAs were examined. In the first derivatives of the melting curves (Fig. 3A), one peak was observed for TP-G1G-36, and two peaks were observed for TP-G2G-37 and TP-G3G-38. The *T*_m_ values obtained from the UV melting curves are shown in Table 1. The *T*_m_ values for TP-G2G-37 (51.7, 76.0 °C) and TP-G3G-38 (49.3, 75.9 °C) were similar to each other, one was higher than that of TP-G1G-36 (66.6 °C), and the other was lower. The UV melting curves for the sub-fragments were also measured (Table 1, Supplemental Fig. S2). The *T*_m_ value of TP-G1G-36 was similar to those of its sub-fragments, TAR-G1G-19 (63.5 °C) and PolyA-17 (62.4 °C). The higher *T*_m_ value of TP-G2G-37 was similar to that of TAR-G2G-22 (73.9 °C) rather than TAR-G2G-21 (69.6 °C). Thus, it was suggested that TP-G2G-37 consists of two independent structural units, TAR-G2G-22 and PolyA-15. This is also true for TP-G3G-38 and its sub-fragments, TAR-G3G-23 and PolyA-15, indicating that A60 stacks to the terminal GC base pair of the TAR stem for TP-G2G-37 and TP-G3G-38. By comparing the *T*_m_ values of the three fragments, it was found that, in the case of TP-G1G-36, the two stems were equally stable, whereas, in the case of TP-G1G-36 and TP-G2G-37, the TAR stems were more stable, and the PolyA stems were less stable.

**Table 1:** *T*_m_ values for the RNA fragments.

Temperature range (°C)	< 60	60–70	> 70
TP-G1G-36		66.6 ± 0.3	
TP-G2G-37	51.7 ± 1.0		76.0 ± 0.4
TP-G3G-38	49.3 ± 3.1		75.9 ± 0.3
TAR-G1G-19		63.5 ± 0.4	
TAR-G2G-21		69.6 ± 0.3	
TAR-G2G-22			73.9 ± 0.5
TAR-G3G-23			74.6 ± 0.2
PolyA-17		62.4 ± 0.2	
PolyA-16	54.6 ± 0.2		
PolyA-15	43.5 ± 0.3		

### Structure analysis by NMR spectroscopy

Figure 3B shows the NMR spectra of the model RNAs with those sub-fragments in the imino proton region. In the case of TP-G1G-36, the imino proton spectrum was almost the sum of the spectra of its sub-fragments except for signals for the joint region including G3 and G105, indicating that the structures of the TAR and PolyA stem loops are similar between the model RNA and its fragments. The G2 signal was not observed for TAR-G1G-19 but was clearly observed for TP-G1G-36, suggesting that the G2-C59 base pair in the TAR stem is stabilized by the PolyA stem. In the case of TP-G2G-37 and TP-G3G-38, many imino proton signals were similar between the model RNAs and those sub-fragments, indicating that similar stem loop structures were formed. However, imino proton signals of base pairs close to the 5′ or 3′ terminals were broad and could not be assigned, suggesting that the structures in the joint region are unstable. In fact, most signals of TP-G2G-37 and TP-G3G-38 broaden with increasing temperature, whereas most of the signals of TP-G1G-36 are stable up to 310 K (Supplemental Fig. S3). Furthermore, the imino ^15^N–^1^H correlation signal for G3 could be observed for G3 labelled TP-G1G-36 but not for TP-G2G-37 and TP-G3G-38 in the ^15^N–^1^H SQC spectra (data not shown). Thus, it is possible that the two stem loops interacted with each other for TP-G1G-36 but not for TP-G2G-37 and TP-G3G-38. It was also indicated with A60-labelled TP-G2G-37 and TP-G3G-38 that the ^13^C–^1^H correlation signals of A60 in these two RNAs were not observed at 283 K, probably due to exchange broadening (data not shown). Then, the solution structure of TP-G1G-36 was determined as described below. For TP-G3G-38, signals due to the four G residues at the 5′ terminus were not clearly observed, and two signals were observed for each of G52-54, suggesting conformational polymorphism in the terminal region (Supplemental Fig. S4). This was also true for TAR-G3G-23.

### Tertiary structure of TP-G1G-36

NMR signals of TAR-G1G-19 and PolyA-17 were assigned by the conventional method, and then, signals of TP-G1G-36 were successfully assigned (Supplemental Fig. S5); signals of imino protons, amino protons of C in the GC base pairs, non-labile protons (H8/H6/H5/H2) of the base and H1′ of ribose were assigned. The signal assignments for the imino proton of G3 and base protons of A60 were confirmed by site-specific labelling with 10% ^13^C/^15^N (Supplemental Fig. S6).

For TP-G1G-36, imino and amino proton signals due to the base pairs of G2-C58 and C59-G105 were observed and an inter-stem NOE between H1 of G2 and H1′ of C59 was observed (Supplemental Fig. S7A). Between G2 and G105, inter-stem NOEs for H8–H8 and H1′–H1 were also observed. An inter-stem NOE may be observed between H8 of G2 and H1′ of G105, which overlaps with the intra-residual NOE between H1′ and H8 of A55 (Supplemental Fig. S7B), resulting in a rather strong NOE peak compared to other intra-residual NOEs between H1′ and H8. Thus, it was found that the TAR and PolyA stems stacked to each other. U106 at the 3′ terminus did not show any NOE with G105, but a weak NOE was observed between H5 of U106 and H8 of G2, suggesting that U106 is located outside of the stacked stems.

**Figure 4:**
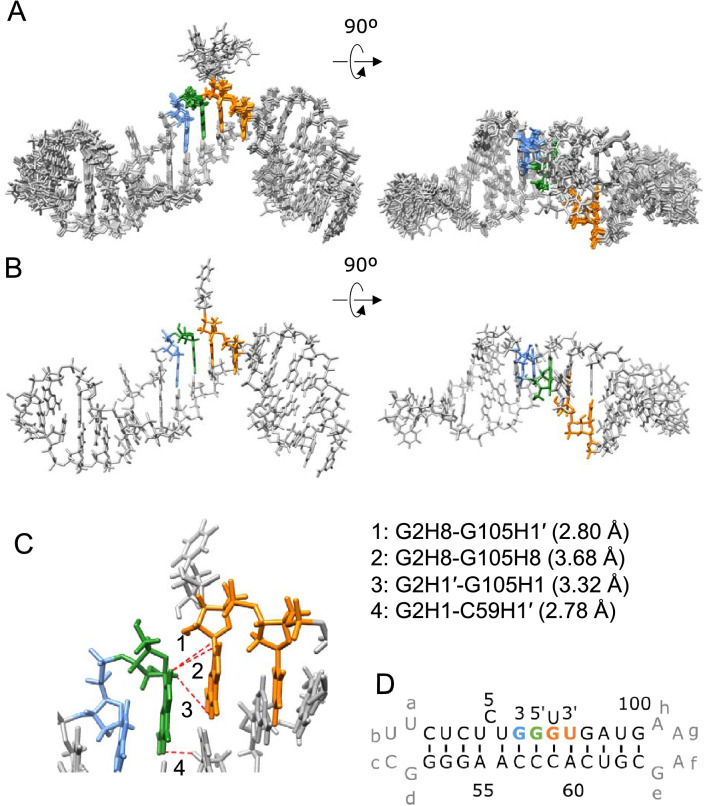
Solution structures of TP-G1G-36. (**A**) Superposition of the 10 accepted structures with the lowest energy. (**B**) The minimized average structures of the 10 structures. The 5′-terminal G, G3 and the 3′-terminal UG residues are indicated by blue, green and orange, respectively. The right panels show the 90-degree rotated views of the left panels. (**C**) Inter-stem NOEs and distances. The proton pairs are indicated by the red lines. The distances derived from the NOEs were shown. (**D**) Secondary structure of TP-G1G-36.

Figure 4 shows the calculated structures of TP-G1G-36 based on the NMR restraints (Supplemental Tables S1, S2). The TAR and PolyA stems form a coaxially stacked stem, and U106 is located outside of the stem. The MD simulation revealed that the coaxially stacked stem was stable and that the conformational dynamics were not affected by the replacement of the 5'-GGU sequence with the Cap structure, 5′-m7GpppGmUm (Supplemental Fig. S8). Notably, the structures of each stem, TAR and PolyA, were similar between the model RNAs and their sub-fragments, except for the terminal regions (data not shown).

## Discussion

**Figure 5:**
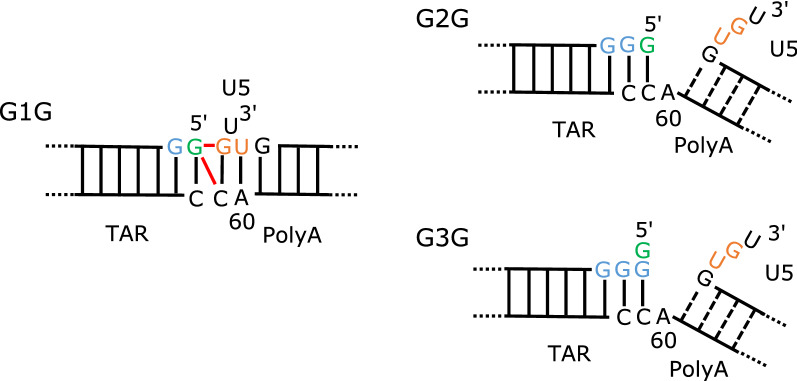
Schematic drawing of the conformational characteristics of the three RNA fragments. The 5′ G residue corresponds to the 5′-Cap. Red lines indicate the observed inter-stem NOEs between the two stems, indicating the formation of the coaxial stem. The same colouring of residues is shown in Fig. 1. The G residues corresponding to the three transcription start sites and 5′-Cap are coloured light blue and green, respectively. U104 and G105 at the 3′-end of the PolyA stem were coloured orange. The A60 residue forms a base pair with U104 in the PolyA region for G1G. It does not pair with the U residue but stacks on the GC base pair at the end of the TAR stem for G2G and G3G. An increase of exposed residues in the U5 region will affect the structure downstream of the U5 region.

The results of the present study clearly showed that the conformation of the model RNAs was affected by the number of G residues at the 5′-terminus (Fig. 5). In the case of G1G, the first G residue, which corresponds to 5′-Cap, forms a stable base pair with the C residue at the joint site between the TAR and PolyA stems, and this GC base pair stacks on the GC base pair at the end of the PolyA stem to form stable coaxial stems. In contrast, for G2G or G3G, the extra GC base pair at the end of the TAR loop prevents the formation of the GC base pair at the end of PolyA to make the two stems structurally independent. As a result, the PolyA stem was destabilized in G2G and G3G. These results agreed with the recent report from Brown et al.^4^. The 3′-flanking residues, U104 and G105 (Fig. 5, orange), are included in the coaxial stem in G1G but exposed from the PolyA stem in the case of G2G and G3G. As proposed by Brown et al.^4^, the structural difference in U104 and G105 may affect the structure of the remaining region of the 5'-UTR. In G1G, incorporation of U104 and G105 into the stable PolyA stem may induce the secondary structure shown in Fig. 1A to from a dimer conformation, and genomic RNA will be incorporated into virions. In contrast, the exposed U104 and G105 and destabilized PolyA stems in G2G and G3G induce formation of the alternative structures in monomeric form and function as mRNAs in the cytosol.

In the present study, we focused on the joint region of TAR and PolyA stems and designed RNA fragments with stable tetraloops. These fragments worked quite well to characterize and emphasize the differences in structure and stability among the three RNAs with different numbers of G residues. Although NMR analysis was performed by using RNAs without the Cap structure, MD simulations indicated that the structure was not affected by the replacement of the terminal GG residues by the Cap structure. It has also been demonstrated that the effect of the Cap moiety on dimerization of the 5′-UTR is similar to that of a phosphodiester-linked 5′ G^2^. It is well known that the Cap structure is critical for its function as an mRNA; thus, the functional difference among the three types of RNAs with different numbers of G residues should be analysed by RNAs with the Cap structure. Nevertheless, the MD simulation supported that the structural differences at the TAR-PolyA junction region among the three RNAs can be evaluated by the model RNA without the Cap structure.

Our previous work showed that the G1 form is dominant in virus particles and that the G3 form is dominant in the cytosol of infected cells^1^, indicating that the G1 form is preferentially packaged into virus particles. The number of G residues of the three RNAs also affected the reverse transcription reaction^1^. An in vitro assay using synthetic HIV-1 RNAs revealed that the abortive forms of minus-strand strong stop cDNA (-sscDNA), which is first synthesized in the reverse transcription reaction, were abundantly generated from G3-form RNA, but dramatically reduced from G1-form RNA^1^. Furthermore, 5′-Cap significantly increased the strand-transfer efficiency of cDNA generated from G1-form RNA^5^. A previous study by Chen and Menees demonstrated that yeast Ty1 retroelement RNA formed a lariat structure through a 2′–5′ bond between the 5′- and 3′-ends of RNA and might play roles in Ty1 reverse transcription at the first strand-transfer step^6^. Thus, it must be elucidated how the structure and/or stability of the TAR-PolyA region affect the reverse transcription reaction as well as packaging. Our model RNA system may work in such analyses as shown in the present work.

## Methods

### Design of RNAs used for structural analyses

Based on the SHAPE analysis^7^ and the predicted secondary structures of the three RNAs in the G1, G2 and G3 forms by vsfold5^8^ and centroid fold^9^, an RNA fragment, TP-G1G-36, was designed (Fig. 2B). TP-G1G-36 consists of a G residue corresponding to 5′-Cap, a truncated TAR stem in the G1 form, a truncated PolyA stem and a U residue with 36 residues in total. The truncated stems were connected by UUCG and GAAA tetraloops. Two related fragments, TP-G2G-37 and TP-G3G-38, with TAR stems in the G2 and G3 forms, respectively, were also designed (Fig. 2B). The secondary structures of the designed fragments were confirmed by vsfold5^8^.

Fragments corresponding to the TAR and PolyA sides of TP-G1G-36, TP-G2G-37 and TP-G3G-38 were also designed (Fig. 2B). In the case of TP-G1G-36, the G residue at the 5′ end (G2), which corresponds to the m^7^G moiety of the 5′-Cap, was assumed to form a base pair with C58. In this case, the sub-fragment for PolyA starts with C59. Therefore, TP-G1G-36 was divided at C58 and C59 to form TAR-G1G-19 and PolyA-17. In the case of TP-G2G-37, the G at the 5′ end (G1) was assumed to form a base pair with C59. In this case, the sub-fragment for TAR starts with A60. Therefore, TP-G2G-37 was divided at C59 and A60 to form TAR-G2G-21 and PolyA-16. In addition, in consideration of the possibility that A60 stacked to the stem on the TAR side, TP-G2G-37 was also divided at A60 and C61 to form TAR-G2G-22 and PolyA-15. In the case of TP-G3G-38, the G residue at the 5′ end (G0), although it is not complementary, was assumed to form a GA base pair with A60 and stacked with the G1-C59 base pair. Therefore, TAR-G3G-38 was divided at A60 and C61 to form TAR-G3G-23 and PolyA-15.

### Preparation of RNA samples

For all RNA fragments, chemically synthesized oligonucleotides were purchased from Hokkaido System Science Co., Ltd. It is noted that the fully ^13^C/^15^N-labelled adenosine residues were incorporated with a content of 10% at position 3 (G3) or 60 (A60) for TP-G1G-36, TP-G2G-37 and TP-G3G-38. The ^13^C/^15^N-labelled phosphoramidite units were purchased from Taiyo Nippon Sanso Co., Ltd., and oligonucleotides were synthesized by Hokkaido System Science Co., Ltd. The labelled RNA fragments were used only for NMR measurements.

The purchased fragments were dissolved in water. For NMR measurement, the counterions were exchanged by the ultrafiltration method: the synthetic RNAs were concentrated by Vivaspin with a molecular weight cut-off value of 3000 (GE Healthcare) and then exchanged in buffer solution.

The purity and conformation of each RNA were checked by native and denatured PAGE, respectively. Gels were stained with SYBR Gold (Thermo Fisher Scientific). Prestain Marker for Small RNA Plus (BioDynamics Laboratory Inc.) was used as the size maker for the denatured PAGE. Only bands for 20 and 30 nt were stained by SYBR Gold.

### UV melting experiment

UV melting curves were measured for the RNA fragments and the melting temperatures (*T*_m_) were obtained. The buffer solution condition of each RNA sample was 20 mM sodium phosphate buffer (pH 6.5) containing 50 mM NaCl. Each sample was prepared as 115 μL with an absorbance at 260 nm of 0.2–0.3 at 25 °C. Melting curves at 260 nm were measured with a V-730BIO UV–Vis spectrophotometer (JASCO corporation) from 25 to 95 °C at 1 degree/min. The measurements were repeated three times for each RNA fragment. The *T*_m_ values were calculated using the program Spectra Manager (JASCO corporation). The *T*_m_ values were confirmed by curve fitting with the second-order function for each peak in the first derivatives of UV melting curves by a homemade program.

### NMR spectroscopy

RNA samples were dissolved in 20 mM sodium phosphate buffer (pH 6.5) containing 50 mM NaCl with 5% D_2_O (99.98 atom%, Taiyo Nippon Sanso).

One-dimensional and 2D spectra, including HOHAHA and NOESY spectra were measured at 283 K. For TP-G1G-36, TP-G2G-37 and TP-G3G-38, imino proton spectra were measured at 283 K, 298 K and 310 K. NMR spectra were measured using an AVANCE-600 spectrometer and analysed with the programs Topspin 3.5 (Bruker BioSpin) and SPARKY^10^.

### Structure determination

The tertiary structure calculation was performed with the conventional method^11^. Distance constraints were derived from the NOE volumes with the mean of the volumes of pyrimidine H5–H6 signals as the measure. For the base pairs for which imino proton signals were observed, plane-related restraints and the hydrogen bonding distance constraints of the base pair were prepared. In addition, for the stem region confirmed by the imino proton signals, dihedral angle restraints for the main chain, sugar packer and rotation around the glycosidic bond were prepared to form the RNA-A conformation. Furthermore, restraints for the C2′-*endo* form based on the intense HOHAHA signal between H1′ and H2′ and for the *syn* form based on the intensities of NOE between H8/H6 and H1′ were generated. The structure calculation was performed by the program CNS (Ver. 1.3, Yale University)^12^ with the protocol described previously^13^. Tertiary structure calculations were performed for TP-G1G-36, TAR-G1G-19, TAR-G2G-22, PolyA-17 and PolyA-15.

### Molecular dynamics simulations

The molecular dynamics (MD) simulation was performed with the program AMBER12^14^. The minimized averaged structure obtained by CNS_SOLVE was used as the Initial model. The charge for the RNA molecule was neutralized by adding sodium ions and the RNA was surrounded by TIP3 water molecules in a box with a buffer distance between the wall of the box and the closest atom in the solute of 9.0 Å. The numbers of sodium ions and water molecules were 35 and 7080, respectively. The equilibration calculation was performed as described previously^13^. The productive simulation in constant volume without positional restraints was performed for 10 ns (10,000,000 steps). The trajectory of the productive simulation was processed by the program ptraj in the AMBER suite^14^ and visualized by the program Chimera^15^.

An RNA with the Cap structure, TP-G1Cap-36, was modelled by replacing the 5′-GGU sequence of TP-G1G-36 with m^7^GpppGmUm by UCSF Chimera. An MD simulation with 10 ns was also performed for TP-G1Cap-36. The numbers of sodium ions and water molecules are 36 and 7075, respectively.

## Supplementary Information

Supplementary Information.

## Data Availability

Atomic coordinates and NMR information for the reported structure have been deposited with the Protein Data bank under accession number 7DD4 and the Biological Magnetic Resonance Data Bank under ID 36393.

## References

[1] Masuda T, Sato Y, Huang YL, Koi S, Takahata T, Hasegawa A, Kawai G, Kannagi M (2015). Fate of HIV-1 cDNA intermediates during reverse transcription is dictated by transcription initiation site of virus genomic RNA. Sci. Rep..

[2] Kharytonchyk S, Monti S, Smaldino PJ, Van V, Bolden NC, Brown JD, Russo E, Swanson C, Shuey A, Telesnitsky A, Summers MF (2016). Transcriptional start site heterogeneity modulates the structure and function of the HIV-1 genome. PNAS.

[3] Menees TM, Muller B, Krausslich HG (2007). The major 5' end of HIV type 1 RNA corresponds to G456,AIDS Res. Hum. Retroviruses.

[4] Brown JD, Kharytonchyk S, Chaudry I, Iyer AS, Carter H, Becker G, Desai Y, Glang L, Choi SH, Singh K, Lopresti MW, Orellana M, Rodriguez T, Oboh U, Hijji J, Ghinger FG, Stewar KT, Francis D, Edwards B, Chen P, Case DA, Telesnitsky A, Summers MF (2020). Structural basis for transcription start site control of HIV-1 RNA fate. Science.

[5] Huang YL, Kawai G, Hasegawa A, Kannagi M, Masuda T (2019). Impact of 50-end nucleotide modifications of HIV-1 genomic RNA on reverse transcription. BBBRC.

[6] Cheng Z, Menees TM (2004). RNA branching and debranching in the yeast retrovirus-like element Ty1. Science.

[7] Watts JM, Dang KK, Gorelick RJ, Leonard CW, Bess JW, Swanstrom R, Burch CL, Weeks KM (2009). Architecture and secondary structure of an entire HIV-1 RNA genome. Nature.

[8] Dawson W, Fujiwara K, Kawai G (2007). Prediction of RNA pseudoknots using heuristic modeling with mapping and sequential folding. PLoS ONE.

[9] Sato K, Hamada M, Asai K, Mituyama T (2009). CENTROIDFOLD: A web server for RNA secondary structure prediction. Nucleic Acids Res..

[10] Goddard, T. D. & Kneller, D. G. *SPARKY 3*. (University of California, San Francisco) (2008).

[11] Sakamoto T, Otsu M, Kawai G (2018). NMR Studies on RNA in Experimental Approaches of NMR Spectroscopy.

[12] Brünger AT, Adams PD, Clore GM, Delano WL, Gros P, Grosse-Kunstleve RW, Jiang J-S, Kuszewski J, Nilges N, Pannu NS, Read RJ, Rice LM, Simonson T, Warren GL (1998). Crystallography and NMR system (CNS): A new software system for macromolecular structure determination. Acta Cryst. D.

[13] Otsu M, Kajikawa M, Okada N, Kawai G (2017). Solution structure of a reverse transcriptase recognition site of a LINE RNA from zebrafish. J. Biochem..

[14] Case DA, Darden TA, Cheatham TE, Simmerling CL, Wang J, Duke RE, Luo R, Walker RC, Zhang W, Merz KM, Roberts B (2012). AMBER 12.

[15] Pettersen EF, Goddard TD, Huang CC, Couch GS, Greenblatt DM, Meng EC, Ferrin TE (2004). UCSF Chimera—A visualization system for exploratory research and analysis. J. Comput. Chem..

