# Supplementary material for: Influence of the 5′-terminal sequences on the 5′-UTR structure of HIV-1 genomic RNA
Source: Sci Rep. 2021 May 25;11:10920. doi: 10.1038/s41598-021-90427-9 (PMC8149415; doi:10.1038/s41598-021-90427-9)
Supplement: Supplementary file 1 — Supplementary Information. [file 41598_2021_90427_MOESM1_ESM.pdf]

Supplemental materials

Influence of the 5'-terminal sequences on the 5'-UTR structure of HIV-1 genomic RNA

Camille Michiko Obayashi[1], Yoko Shinohara[2], Takao Masuda[3] and Gota Kawai[1,2,*]

[1] Department of Life Science, Graduate School of Advanced Engineering, Chiba Institute of Technology, Tsudanuma, 2-17-1, Narashino-shi, Chiba, 275-0016, Japan
[2] Department of Life and Environmental Sciences, Graduate School of Engineering, Chiba Institute of Technology, Tsudanuma, 2-17-1, Narashino-shi, Chiba, 275-0016, Japan
[3] Department of Immunotherapeutics, Graduate School of Medical and Dental Sciences, Tokyo Medical and Dental University, Yushima, 1-5-45, Bunkyo-ku, Tokyo, 113-8519, Japan
* To whom correspondence should be addressed. Tel: +81-47-478-0425 Fax: +81-47-478-0425; Email: gota.kawai@p.chibakoudai.jp

Contents

Supplemental Figure S1. Comparison of TP-G1G-36 and TP-G1Cap-36 for TAR and PolyA regions
Supplemental Figure S2. The first derivatives of the melting curves
Supplemental Figure S3. Temperature dependences of imino proton signals
Supplemental Figure S4. Possible secondary structures of TP-G3G-38
Supplemental Figure S5. Sequential assignment of TP-G1G-36
Supplemental Figure S6. NMR spectra of TP-G1G-36 with A60-specific $^{13}C/^{15}N$ labelling
Supplemental Figure S7. Inter-stem NOEs observed for TP-G1G-36
Supplemental Figure S8. Results of MD simulations

Supplemental Table S1. NMR constraints and structural statistics for TP-G1G-36
Supplemental Table S2. NMR constraints for each residue of TP-G1G-36

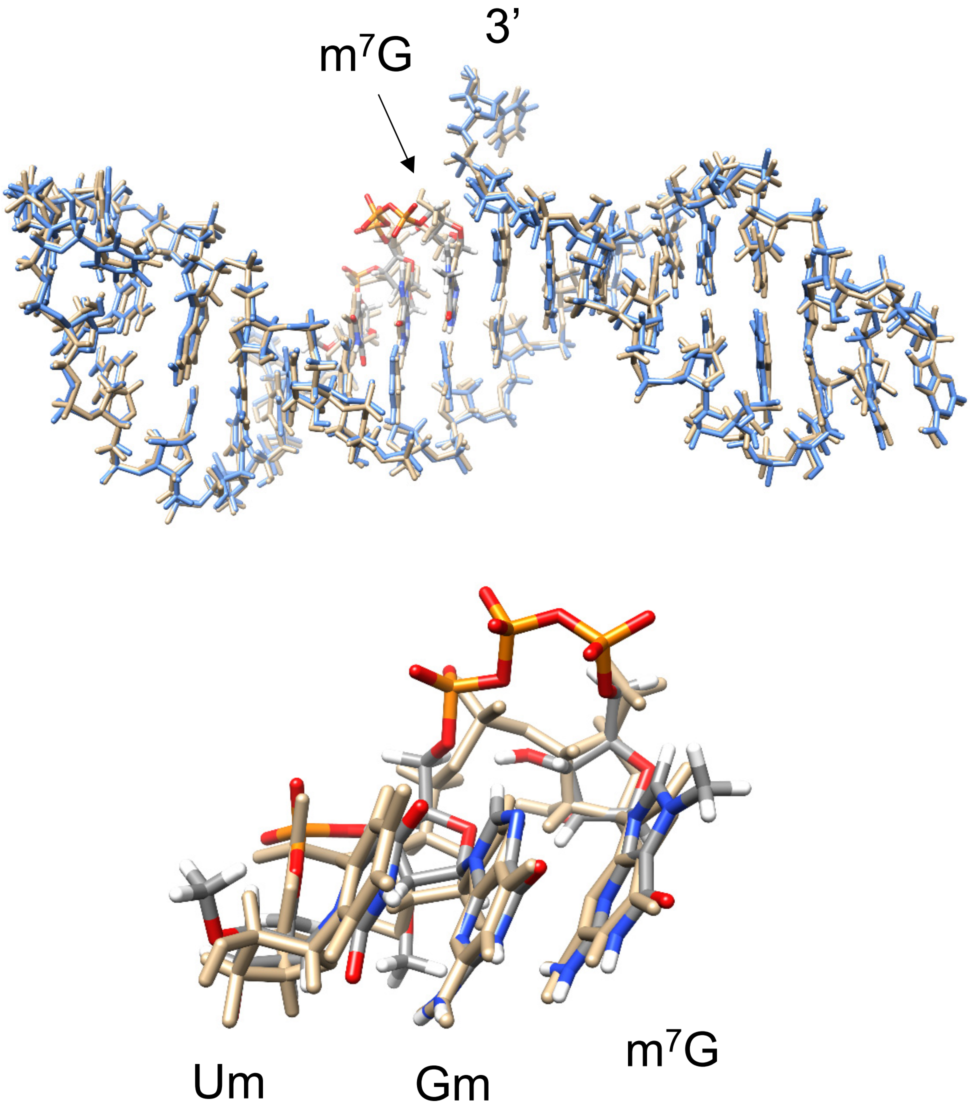

Supplemental Figure S1. Comparison of TP-G1G-36 and TP-G1Cap-36

The minimized-averaged structures obtained in this study was used as the initial structure of the MD simulation. An RNA with the Cap structure, TP-G1Cap-36, was modelled by replacing the 5'-GGU sequence of TP-G1G-36 with m7GpppGmUm. The structures after the equilibration for the MD simulation were superposed. In general, the structures were maintained during a 10 ns MD simulation as shown in Fig. S6.

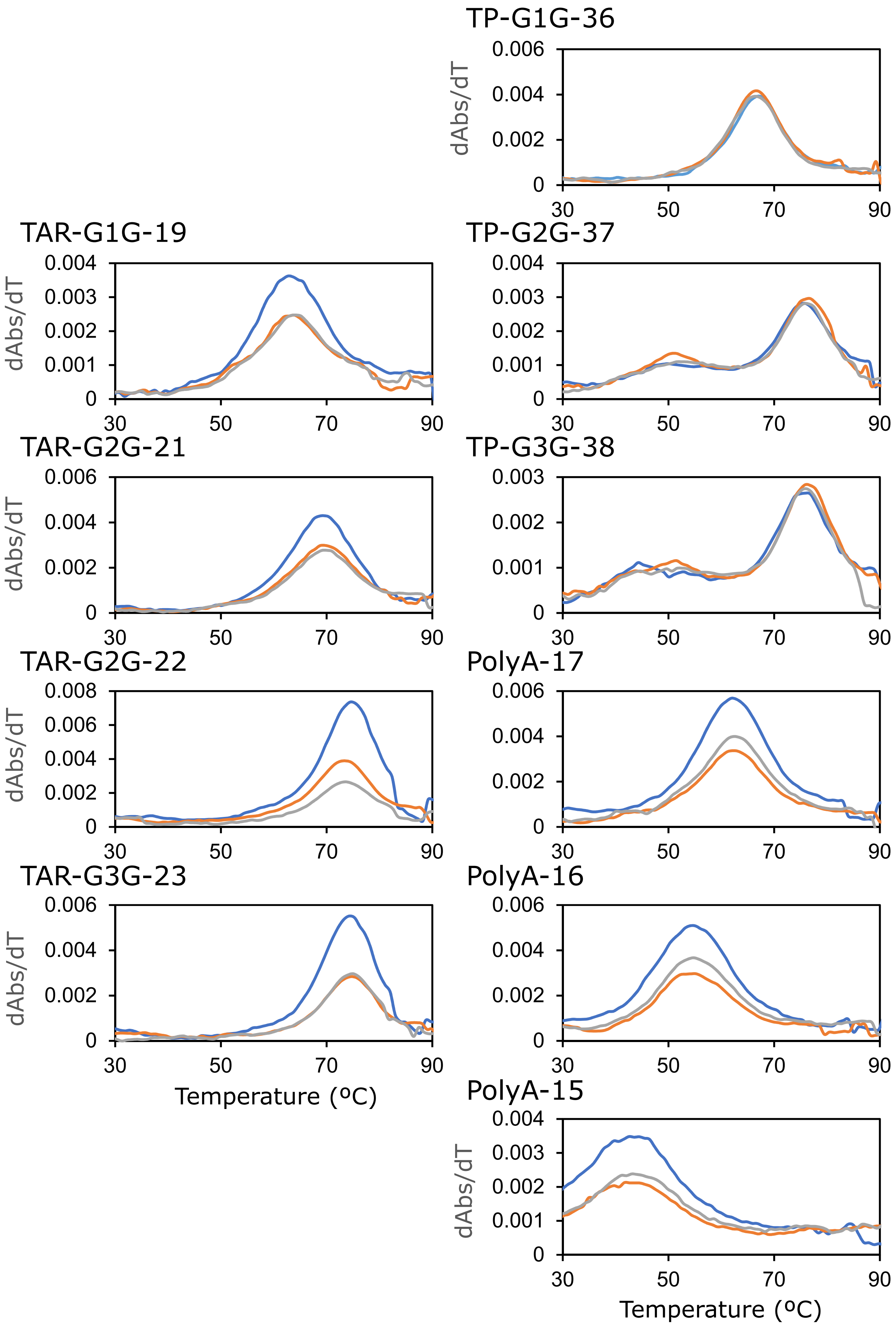

Supplemental Figure S2. The first derivatives of the melting curves

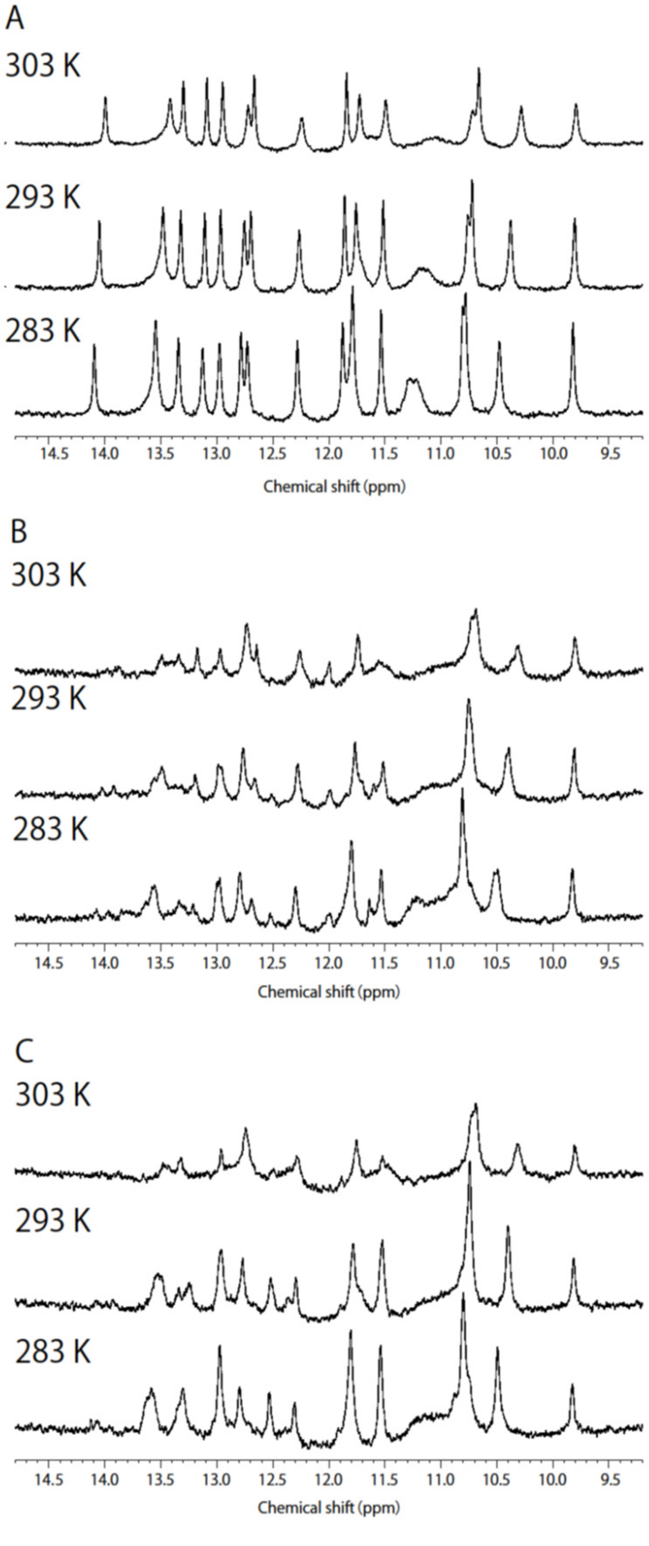

Supplemental Figure S3. Temperature dependences of imino proton signals
A: TP-G1G-36, B: TP-G2G-37, C: TP-G3G-38

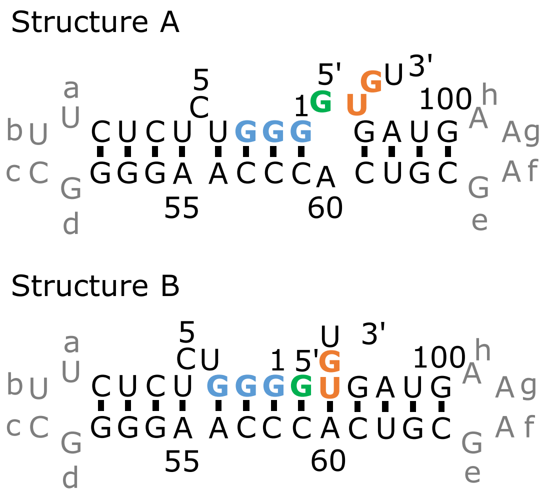

Supplemental Figure S4. Possible secondary structures of TP-G3G-38

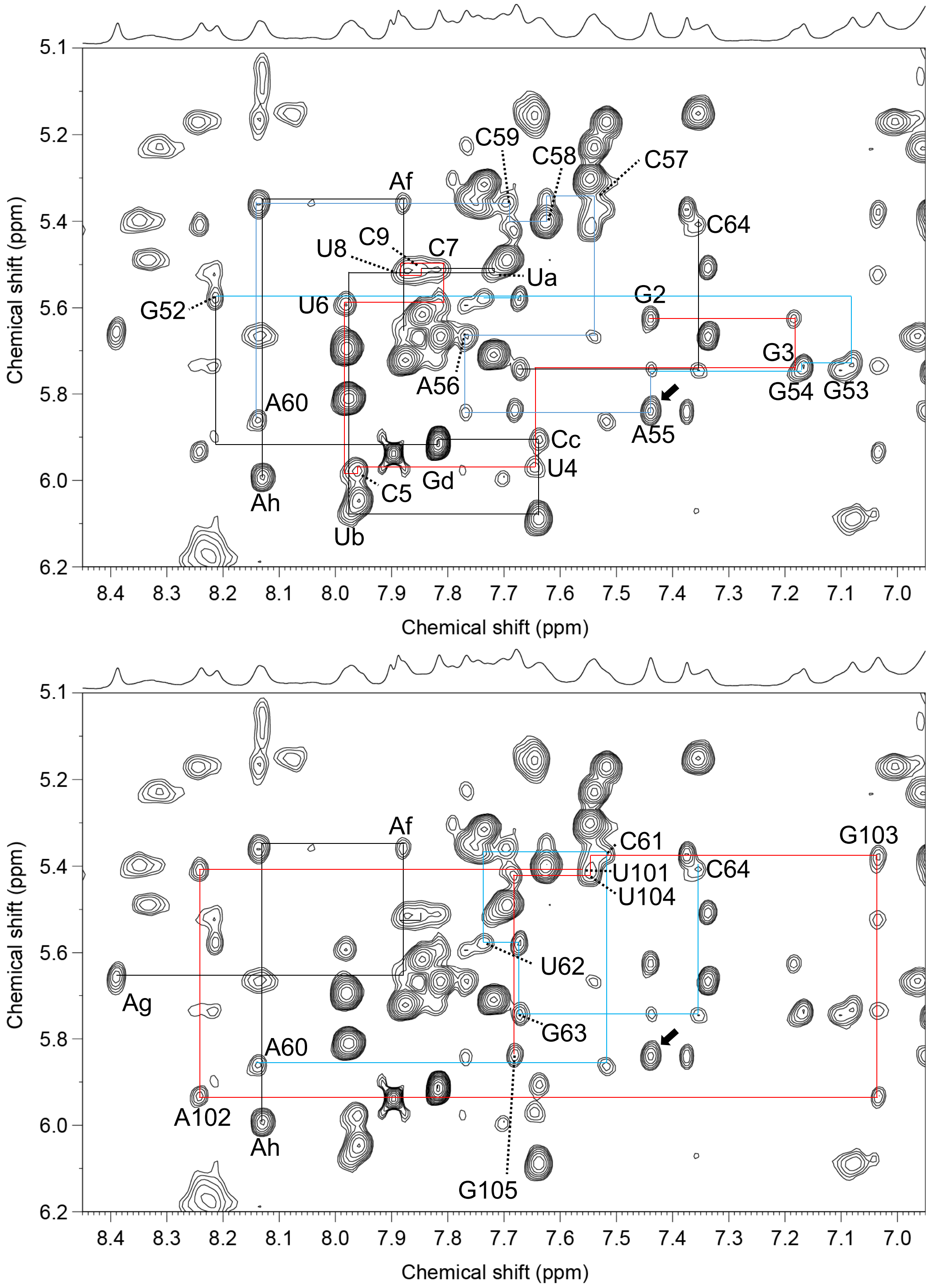

Supplemental Figure S5. Sequential assignment of TP-G1G-36 for TAR (upper) and PolyA (lower) regions

A portion of NOESY spectrum with signal assignment was shown. The block arrow indicates the inter-stem NOE between H1' of G105 and H8 of G2. It is noted the H1' of G100 resonates at 3.619 ppm which is outside of the region shown.

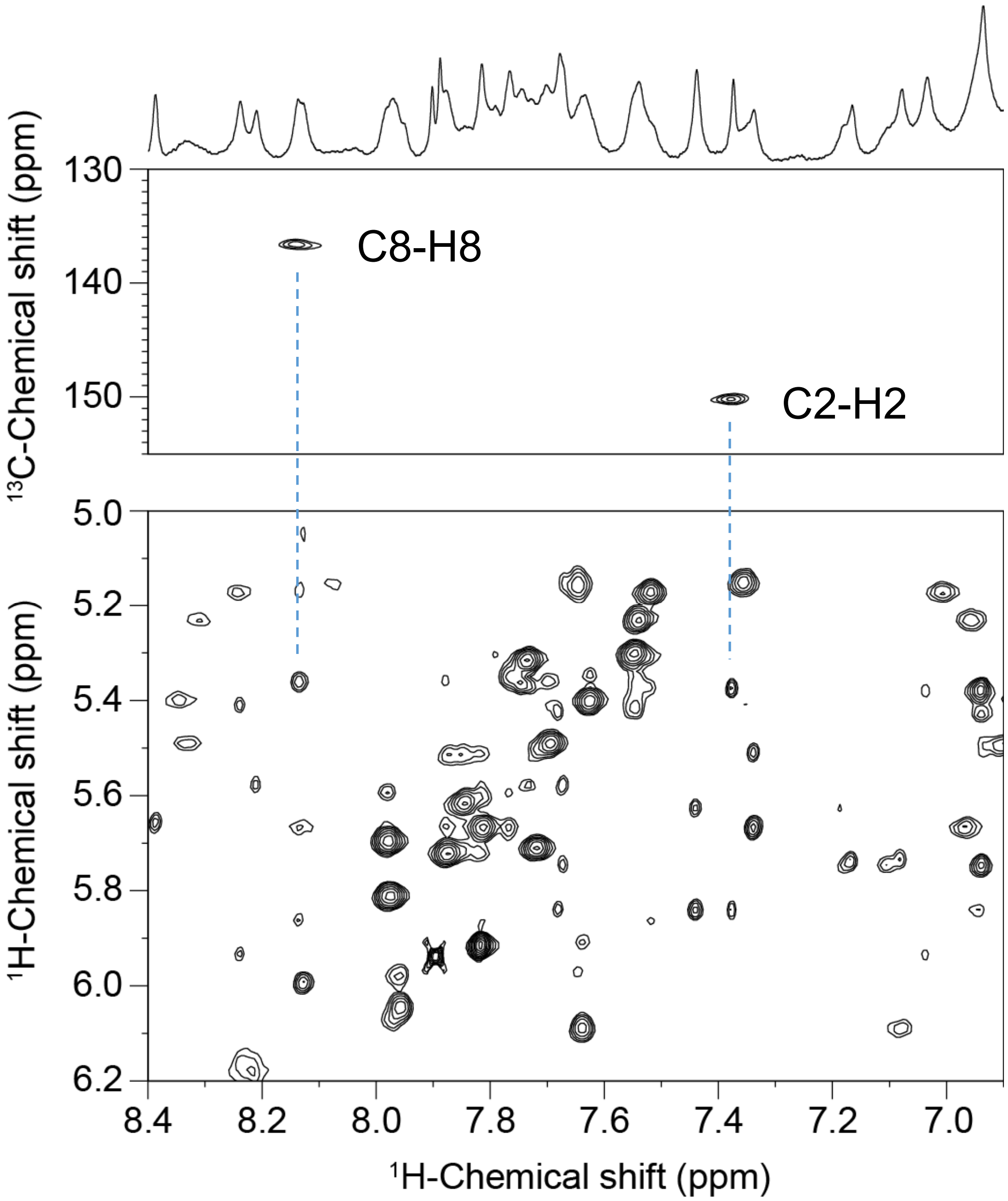

Supplemental Figure S6. NMR spectra of TP-G1G-36 with A60-specific $^{13}C/^{15}N$ labelling

Upper: ^{13}C-^{1}H SQC spectrum of A60 10% labelled TP-G1G-36. Lower: NOESY spectrum of the same RNA. By using the 10% labelled sample, the resonances of the labelled residue were successively assigned. It is noted that SQC signals for TP-G2G-37 and TP-G3G-38 were not observed due to the broadening.

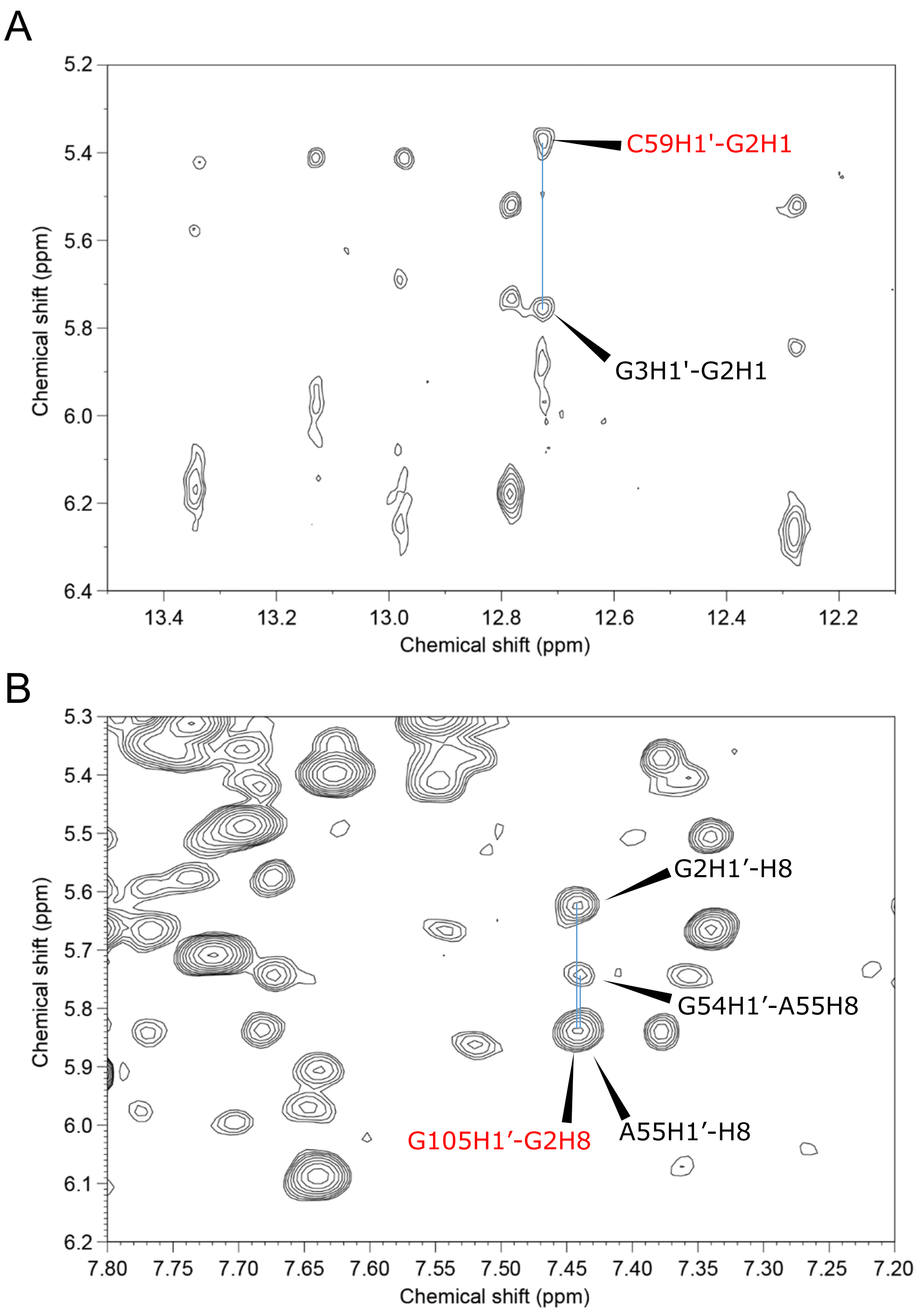

Supplemental Figure S7. Inter-stem NOEs observed for TP-G1G-36 A: An inter-stem NOE between H1 of G2 (TAR stem) and H1' of C59 (PolyA stem). B: An inter-stem NOE between H8 of G2 (TAR stem) and H1' of G105 (PolyA stem), An intra-residual NOE between H1' and H8 of A55 was overlapped to the inter-stem NOE to give a rather strong peak.

TP-G1G-36

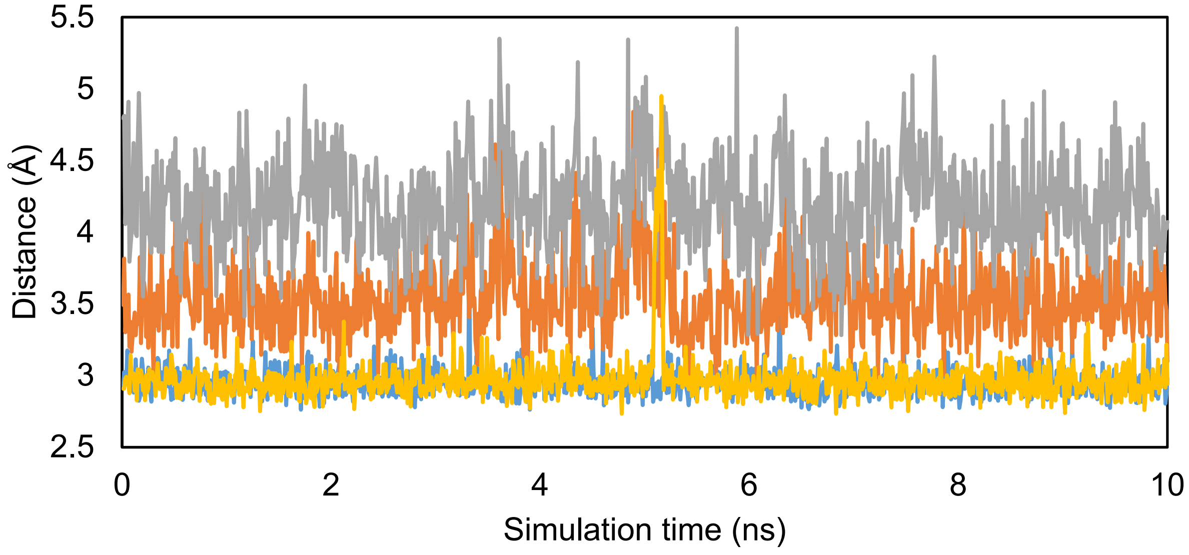

TP-G1Cap-36

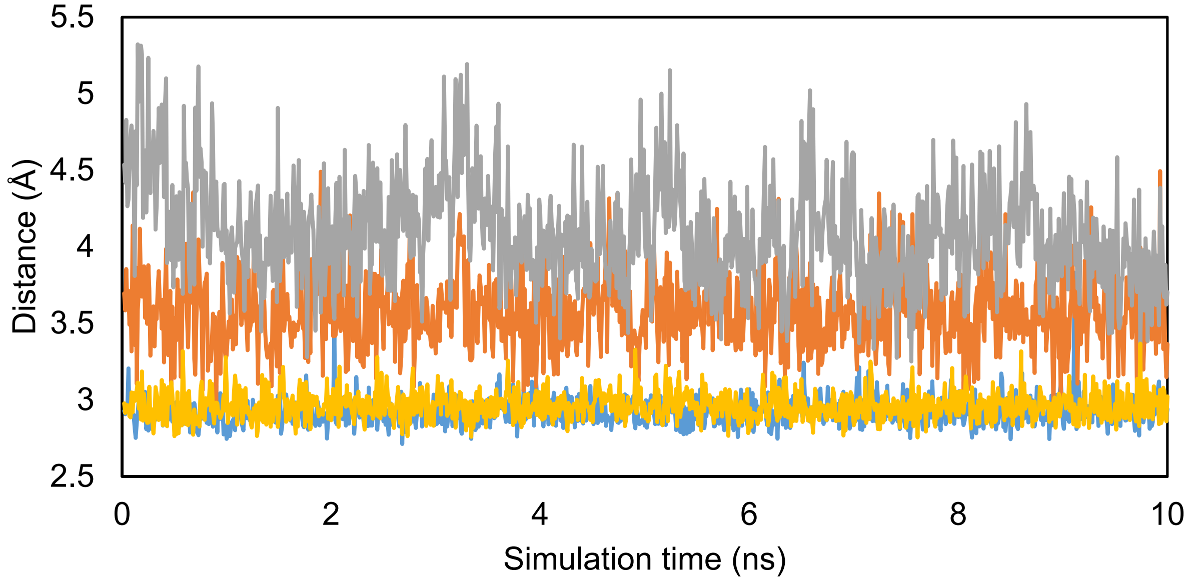

Supplemental Figure S8.Results of MD simulations
Distances between imino nitrogen atoms for the terminal GC base pairs, G2-C58 and C59-G105, as well as for the inter-stem bases, G2-C59 and G2-G105.

Supplemental Table S1. NMR constraints and structural statistics for TP-G1G-36

Number of experimental restraints	
Distance restraints	289
Intraresidue	77
Sequential	78
Medium range	17
Long range	45
vdw distance	36
Hydrogen bonding	36
Dihedral restraints	305
Planarity for base pairs	14
Heavy-atoms r.m.s. deviation (Å)[a]	
All	1.296 ± 0.506
All (pairwise)	1.419 ± 0.262
Backbone	1.049 ± 0.426
Backbone (pairwise)	1.080 ± 0.239
TAR stem (2-4,6-9,14-20)	0.388 ± 0.156
TAR stem (pairwise)	0.460 ± 0.091
PolyA stem (21-26,31-36)	0.460 ± 0.162
PolyA stem (pairwise)	0.291 ± 0.119
r.m.s.d. around the ideal values	
bonds (Å)	0.0030 ± 0.00005
angle (°)	0.6770 ± 0.0096

[a]Averaged r.m.s.d. between an average structure and the 10 converged structures were calculated. The converged structures did not contain experimental distance violation of >0.5 Å or dihedral violation >5° .

Supplemental Table S2. NMR constraints for each residue of TP-G1G-36

	Distance restraints			Dihedral restraints		
	intra residual NOEs	inter residual NOEs*	vdw distances*	Sugar pucker from HOHAHA	Backbone for RNA-A	*syn-anti*
G2	8	11	1	5	3	1
G3	1	9	0	5	5	1
U4	3	1	1	5	2	1
C5	3	1	0	5	0	1
U6	1	3	2	5	3	1
C7	0	8	3	5	5	1
U8	1	6	1	5	5	1
C9	0	6	2	5	2	1
U10	5	16	4	5	0	1
U11	5	6	4	5	0	1
C12	16	11	4	5	0	1
G13	2	9	1	0	0	1
G14	2	7	2	5	3	1
G15	2	7	1	5	2	1
G16	2	14	0	5	3	1
A17	0	10	2	5	5	1
A18	1	6	4	5	5	1
C19	0	7	2	5	5	1
C20	1	7	2	5	2	1
C21	1	9	2	5	3	1
A22	2	10	2	5	5	1
C23	1	10	4	5	5	1
U24	1	5	6	5	5	1
G25	1	10	3	5	5	1
C26	2	8	0	5	2	1
G27	1	1	0	5	0	1
A28	3	6	0	5	0	1
A29	1	9	2	5	0	1
A30	2	5	2	5	0	1
G31	1	12	0	5	3	1
U32	1	10	0	5	5	1
A33	5	10	2	5	5	1
G34	1	14	2	5	5	1
U35	0	7	5	0	5	1
G36	1	9	6	5	2	1
U37	0	0	0	5	0	0
total	77	280	72	170	100	35

*inter-residual restraints were counted twice.
